# Supplementary material for: Allosteric regulation by c-di-AMP modulates a complete N-acetylglucosamine signaling cascade in Saccharopolyspora erythraea
Source: Nat Commun. 2024 May 7;15:3825. doi: 10.1038/s41467-024-48063-0 (PMC11076491; doi:10.1038/s41467-024-48063-0)
Supplement: Supplementary file 1 — Supplementary Information [file 41467_2024_48063_MOESM1_ESM.pdf]

Supplementary Information:

Allosteric regulation by c-di-AMP modulates a complete N-acetylglucosamine signaling cascade in *Saccharopolyspora erythraea*

Di You[1]*, Liu-Chang Zhao[1], Yu Fu[1], Zhi-Yao Peng[1], Zong-Qin Chen[1], Bang-Ce Ye[1,2]*

[1]Lab of Biosystems and Microanalysis, State Key Laboratory of Bioreactor Engineering, East China University of Science and Technology, Shanghai 200237, China

[2]Institute of Engineering Biology and Health, Collaborative Innovation Center of Yangtze River Delta Region Green Pharmaceuticals, College of Pharmaceutical Sciences, Zhejiang University of Technology, Hangzhou 310014, Zhejiang, China

* Corresponding authors

Corresponding authors

Di You
Email: 030111115@mail.ecust.edu.cn

Bang-Ce Ye
Email: bcye@ecust.edu.cn

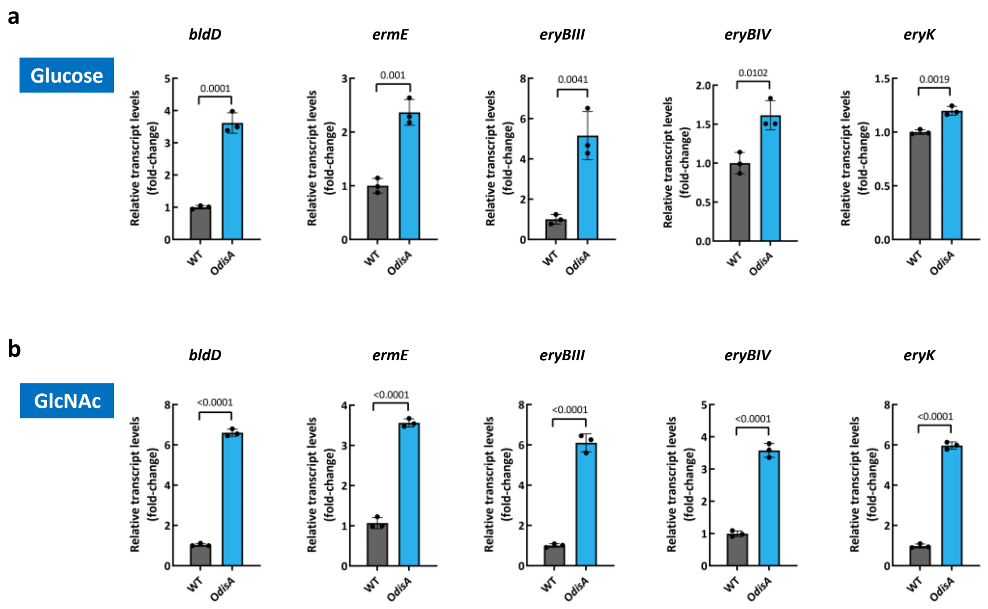

Supplementary Figure 1. The transcription levels of the known genes critical for development and antibiotic production in *S. erythraea. S. erythraea* WT and O*disA* strains were grown till late exponential growth (48 h) in liquid TSB medium with glucose (**a**) or GlcNAc (**b**) addition. Fold change represents the expression level compared to the WT strain. Data are presented as mean values ± SD for n = 3 biological replicates. An unpaired two-sided t test was used for the statistical analysis. Source data are provided as a Source Data file.

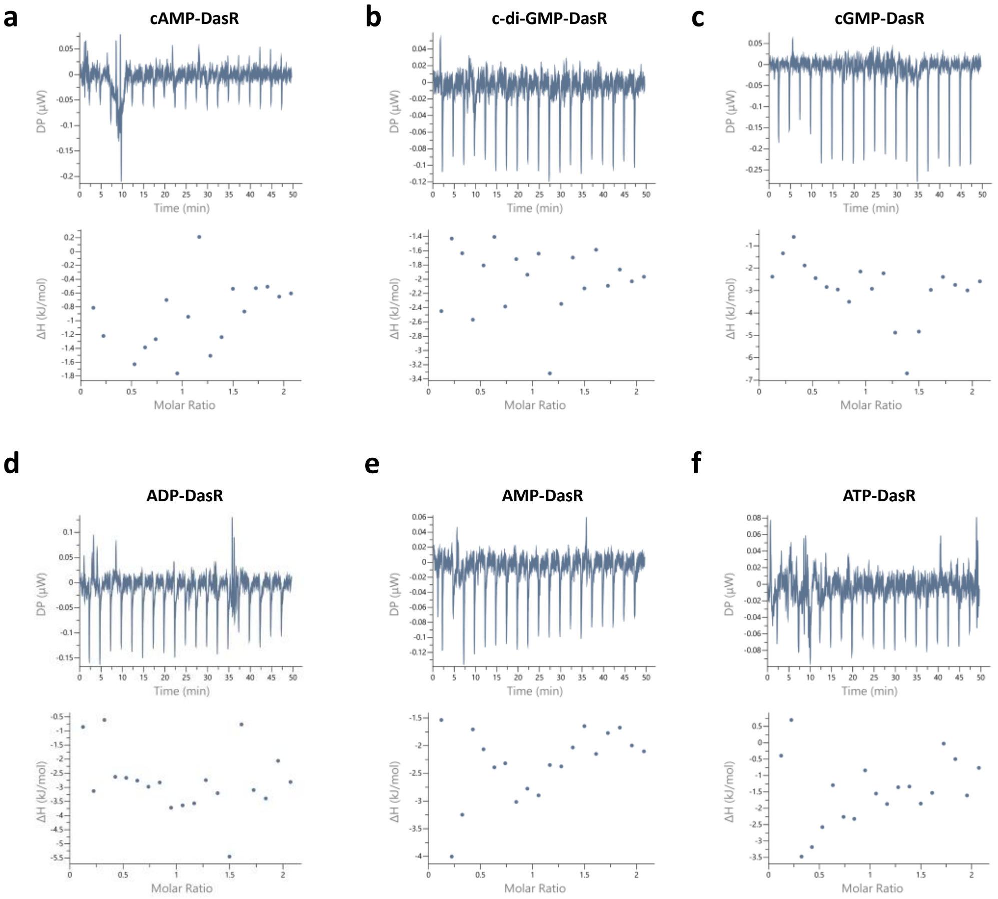

Supplementary Figure 2. Characterization of the interactions of nucleotides with DasR using ITC. Titration of nucleotides (300 μM) into DasR (30 μM). ITC measurements of DasR titrated with cAMP (**a**), c-di-GMP (**b**), cGMP (**c**), ADP (**d**), AMP (**e**), and ATP (**f**).

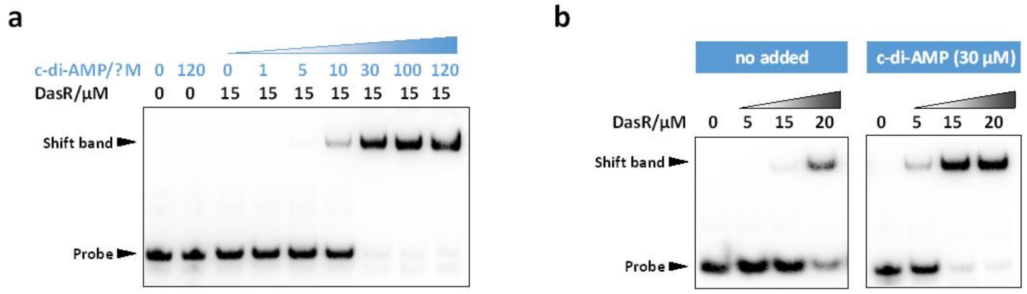

Supplementary Figure 3. EMSA of DasR binding to its target gene *nagA* promoter. **a** Purified His-DasR (15 μM) and DNA fragment were incubated with a gradient concentration of c-di-AMP (1, 5, 10, 30, 100, and 120 μM). **b** Purified His-DasR and DNA fragment were incubated without c-di-AMP (left) or with 30 μM c-di-AMP (right). Representative pictures of two independent experiments with similar results are shown. Source data are provided as a Source Data file.

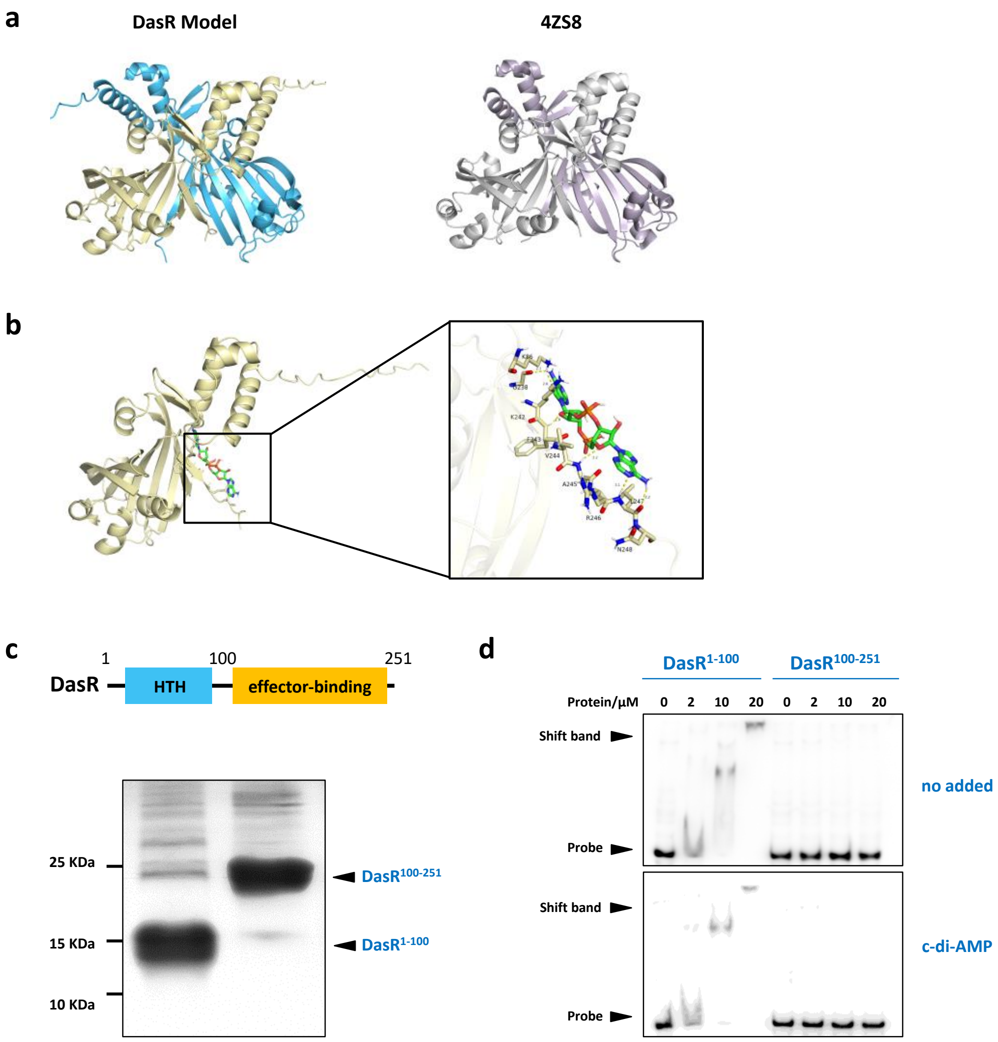

Supplementary Figure 4. The putative c-di-AMP-DasR complex. **a** Model of *S. erythraea* DasR dimer obtained from Alphafold2 compared with *S. coelicolor* DasR (PDB; 4ZS8). **b** The putative DasR-c-di-AMP docking (ranking No.1 with a binding energy of -6.81 kcal/mol). The close-up shows the predicted interaction sites, annotated with the putative binding residues. Interactions are denoted with labels. **c** Construction of the DasR HTH (DasR$^{1-100}$) and DasR EB (DasR$^{100-251}$) mutants. Representative pictures of two independent experiments with similar results are shown. **d** EMSA of DasR mutants binding to its target gene *nagA* promoter. Purified His-DasR mutants and DNA fragment were incubated with 100 μM c-di-AMP (bottom) or without c-di-AMP (top). Representative pictures of two independent experiments with similar results are shown. Source data are provided as a Source Data file.

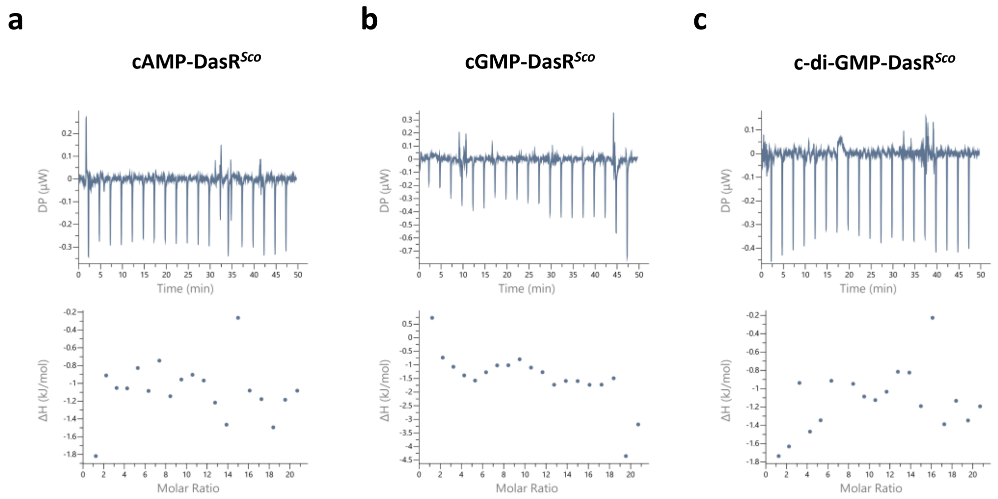

Supplementary Figure 5. Characterization of the interactions of nucleotides with DasR^{Sco} using ITC. Titration of nucleotides (1 mM) into DasR^{Sco} (10 μM). ITC measurements of DasR^{Sco} titrated with cAMP (**a**), cGMP (**b**), and c-di-GMP (**c**).

Supplementary Table 1. Secondary structural elements in DasR

Protein	Helix (%)	Antiparallel (%)	Parallel (%)	β-turn (%)	Random coil (%)	Total sum (%)
**DasR**	99.1	0	0.2	4.0	0.4	103.6
**DasR + c-di-AMP**	88.6	0.1	1.2	8.7	2.7	101.3

Supplementary Table 2. Strains and plasmid

Strains or plasmids	Reference or source
**Strains**	
S. erythraea NRRL23338	DSM 40517
S. erythraea Δ*dasR*	(21)
S. erythraea CdasR	(21)
S. erythraea Δ*dasR*::*disA*	This work
S. erythraea OdasR	This work
S. erythraea OdisA	This work
E. coli DH5α	TransGen Biotech
E. coli BL21(DE3)	TransGen Biotech
**Plasmids**	
pIB139	(44)
pET-28a	Thermo Scientific

Supplementary Table 3. Primers used in this work

Primers for overproduction of proteins

Oligonucleotides	Sequence (5 to 3`)
DasR (SACE_0500)	CCTGGTGCCGCGCGGCAGCCATATGATCGGTGCCAAGCGGGGCGGAGAAC
	GGTGCTCGAGTGCGGCCGCAAGCTTGCCGCAAGCTTTCAGGCGGGCGGGTTGAG
DasR$^{1-100}$	AGCAAATGGGTCGCGGATCCCTCGAAACATCGGTGCCAAGC
	CGAGTGCGGCCGCAAGCTTGCTGGAGCCGCTGGGCG
DasR$^{100-251}$	AGCAAATGGGTCGCGGATCCCTCACCGGCTACACCGAGG
	CGAGTGCGGCCGCAAGCTTGGGCGGGCGGGTTGAGG
DisA (SACE_0435)	CCTGGTGCCGCGCGGCAGCCATATGGTTAATGAAAAACTGCGTGCAACCTTAGC
	GGTGCTCGAGTGCGGCCGCAAGCTTTTATGCATAGCGATCCATAATACTTGCTTCG
DasR^{Sco} (SCO5231)	CCTGGTGCCGCGCGGCAGCCATATGATGAGCACCGACGTCAGCAGTGC
	GGTGCTCGAGTGCGGCCGCAAGCTTCTAGTCCTGGGGCCGCTTGAGGCG

Primers for EMSA with biotin labeling

Primer name	Sequence (5 to 3`)
Universal primer	Biotin-AGCCAGTGGCGATAAG
SACE_0435	AGCCAGTGGCGATAAGCGGCCTGACCCAGGACATCACCAC
	AGCCAGTGGCGATAAGCGGGCTCACCCACCCGTCGGCATGT
nagA	AGCCAGTGGCGATAAGGCCCTGCAACGGATC
	AGCCAGTGGCGATAAGCGCATGTGAGGTCACG
SCO3351	AGCCAGTGGCGATAAGGACAGGAAGCTGTTCTTCCGTGGCG
	AGCCAGTGGCGATAAGGAGCGCGGCCCGGCGTTGCGGGGTG

Primers for RT-PCR

Primer name	Sequence (5 to 3`)
RT-0435F	ACGGTGCGGTGGTGCTC
RT-0435R	CCGACAGGGTCTGGGCG
RT-*nagF*F	GGTCAGCGACGGGGCGAAGGT
RT-*nagF*R	TGTCCTCGGCCAGCGAGTCGG
RT-*nagK*F	GCGGGGCATGGGCGTCTACT
RT-*nagK*R	CGATGGGCATCACGATCACGT
RT-*nagA*F	GCGGGGCATGGGCGTCTACT
RT-*nagA*R	CGATGGGCATCACGATCACGT
RT-*nagB-II*F	AGGTGCGGACATCTGCGCCATC
RT-*nagB-II*R	GCGGGTCCGGGTCACCTTCTTG
RT-*bldD*-F	GGTCGTCGGGTCCTATGAAC
RT-*bldD*-R	GCTCCAGGTTGATCACGACT
RT-*ermE*-F	CCTCCAGGCACCAGTCCAC
RT-*ermE*-R	AGTCGTTGCGGGAGAAGCT
RT-*eryBIII*-F	GTCCCGCTCGAACTGGTCAAGT
RT-*eryBIII*-R	TGGAGTCGTTGCTGCCGATGTC
RT-*eryBIV*-F	GCAGCCGCAGGATCACGC
RT-*eryBIV*-R	GCCGCCCGTGTTGCTCTA
RT-*eryK*-F	CCGATGGACCACGAGCAGTT

RT-*eryK*-R	AAGGCGGGAGATCAGGTCGT
RT-8101F	CAAAGGAATTGACGGGGGC
RT-8101R	CACGGGGTCGAGTTGCAGA
