# Supplementary material for: Allosteric regulation by c-di-AMP modulates a complete N-acetylglucosamine signaling cascade in Saccharopolyspora erythraea
Source: Nat Commun. 2024 May 7;15:3825. doi: 10.1038/s41467-024-48063-0 (PMC11076491; doi:10.1038/s41467-024-48063-0)
Supplement: Supplementary file 3 — Description of Additional Supplementary Files [file 41467_2024_48063_MOESM3_ESM.docx]

**File name: Supplementary Data 1**

Description: S. erythraea DasR dimer.

**File name: Supplementary Data 2**

Description: DasR-c-di-AMP docking.
